# Editorial: Insights in exercise physiology: 2021

**DOI:** 10.3389/fphys.2022.975731

**Published:** 2022-07-14

**Authors:** Giuseppe D'Antona, Martin Burtscher

**Affiliations:** ^1^ CRIAMS-Sport Medicine Centre Voghera, University of Pavia, Pavia, Italy; ^2^ Department of Public Health, Experimental and Forensic Medicine, University of Pavia, Pavia, Italy; ^3^ Department of Sport Science, Medical Section, University of Innsbruck, Innsbruck, Austria

**Keywords:** molecular mechainsm, exercise, adaptations, Frontiers, challenges

The roots of exercise physiology date back into antiquity, when Susruta (about 600 B.C.) in India was likely the first physician to prescribe moderate daily exercise, and Hippocrates (460–370 B.C.) in Greece was the first to provide a written exercise prescription, and Galen (129–210 A.D.) recommended to include regular physical activity in the management of avoiding illness (Tipton, 2014). While physiological concepts at that time were far from current understanding, it were findings from seminal research work published in the first three decades of the 20th century that laid the foundations of modern exercise physiology (Lindinger, 2022). For instance, August Krogh (regulation of oxygen supply to working muscles) and Archibald Vivian Hill (production of heat in the muscle and concept of maximal oxygen uptake) pioneered contemporary exercise physiology, both being awarded the Nobel Prize in Physiology or Medicine in 1920 and 1922, respectively. Subsequently hundreds of exercise laboratories have been set up around the world and thousands of publications contributed and still contribute to a more complete understanding of exercise physiology. In particular, during the past few decades, significant advances have been made in analytical laboratory techniques, where the fields of biochemistry, genetics and molecular biology pushed forward exercise science into a new era (Gomes et al., 2020).

Part of the current scope of research in exercise physiology is represented by 11 papers contributing to the Research Topic “Insights in Exercise Physiology: 2021,” from different areas.

## Cardiovascular exercise physiology

It is widely accepted that opportunely dosed exercise has the potential to treat chronic cardiovascular diseases, including coronary artery disease (CAD), heart failure, and hypertension but several open issues remain on the mechanisms underlying the cardiac and vascular benefits, the optimization and individualization of exercise type, intensity, and duration, and the identification of the limiting factors of the exercise tolerance in patients. Overall, studies published in the present Research Topic shed new light onto some important aspects of the field including the vascular response to exercise in the presence of heart failure/CAD and the role of innovative rehabilitation approaches in cardiopathic patients.

Mannozzi and colleagues examined whether baroreflex dysfunction in heart failure exacerbated ventricular-vascular uncoupling at rest, and during exercise in response to baroreceptor unloading by performing bilateral carotid occlusions in chronically instrumented conscious canines (Mannozzi et al.). The findings indicate that the already challenged orthostatic and exercise tolerance is impaired by the enhanced ventricular-vascular uncoupling during baroreceptor unloading, negatively affecting exercise tolerance and quality of life in heart failure patients (Mannozzi et al.).

Montalvo and colleagues evaluated exercise-induced blood flow patterns in the carotid artery during various types of exercise (maximal tests on a treadmill, cycle-ergometer, and arm-ergometer, and 1-repetition maximum tests of the squat, bench press, and biceps curl) at 3 different intensities (low, moderate, high) (Montalvo et al.). Simultaneous real-time ultrasound image and blood flow of the carotid artery showed that all exercise intensities across all modalities resulted in turbulent blood flow. However, endothelial shear stress (ESS) was greatest during treadmill at a high intensity, while bench press and biceps curls yielded the least ESS (Montalvo et al.).

Fanget and colleagues proved tele-rehabilitation as effective and safe alternative for cardiac rehabilitation during the COVID-19 period, suggesting this approach also to be used to facilitate the continuity of care for patients unable to participate in center-based cardiac rehabilitation (Fanget et al.).

Labeix and colleagues demonstrated benefits of a specific inspiratory muscle training (IMT) during cardiac rehabilitation of patients suffering from coronary artery disease with moderate obstructive sleep apnea (Labeix et al.). Additional IMT resulted in a decrease of the apnea-hypopnea index in those patients.

## Molecular adaptations to exercise

Despite the tremendous advancements in the knowledge of the molecular modifications underlying the musculoskeletal plastic response to exercises, many aspects deserve further study. In particular, doubts still exist about the fine-tune mechanisms triggering the underlie response based on variations in exercise quality, intensity, duration and volume and their countless potential combinations, both in health and disease. In the present RT three works investigated fundamental molecular aspects of the individual response to training including the impact of volume and intensity onto the biochemical muscular adaptations, the role of miRNAs in the individual response to exercise, and the significance of mitochondrial regulator protein cyclophilin-D, involved in cell death due to ischemia/reperfusion injury, in the adaptative response to exercise.

Vann and colleagues studied the effects of higher-load (HL) versus (lower-load) higher-volume (HV) resistance training on skeletal muscle hypertrophy, strength, and muscle-level molecular adaptations in young men (Vann et al.). Single leg training was performed over 6 weeks. Cross-sectional area of the vastus lateralis muscle was only increased after HV, but HL improved leg extensor strength more than HV (Vann et al.). Biochemical assays revealed that integrated non-myofibrillar protein synthesis rates were higher in HV compared to HL.

Witvrouwen and colleagues evaluated changes in plasma-derived miRNAs by acute and chronic exercise in heart failure with reduced ejection fraction (HFrEF) in order to assess whether these can mechanistically be involved in the variability of exercise-induced adaptations (Witvrouwen et al.). The authors demonstrated some interesting relationships between miRNAs and exercise responses, e.g., baseline miR-23a predicted VO_2_peak response to training.

Radhakrishnan and colleagues investigated whether acute cyclophilin-D (Cyp-D) ablation, using tamoxifen-induced ROSA26-Cre-mediated, would downregulate oxygen consumption and trigger an adaptive response that manifests in higher exercise efficiency in conditional knockout mice (Radhakrishnan et al.). The authors demonstrated (as previously shown for constitutive Cyp-D ablation) that acute Cyp-D ablation also induces a state of increased O_2_ utilization efficiency, associated with a metabolic switch toward preferential utilization of glucose via AMPK-TBC1D1 signaling nexus (Radhakrishnan et al.).

## Exercise injury and recovery

In the field of exercise physiology researchers often focus on the study of the mechanisms that underlie the appearance of exercise-induced injuries. The latter must be considered in the frame of the complexity and peculiarity of the exercise gesture and often include problems that go far beyond the simple musculoskeletal and tendon involvements. In fact, new research horizons appear in relation to the identification of the mechanisms of exercise-induced damage, both at the gastrointestinal and renal levels. All these aspects have an obvious great practical impact in the context of the physical preparation and rehabilitation.

In the present RT, Kirsten Legerlotz and Tina Nobis comprehensively dealt with the complexity regarding effects of fluctuating female hormones on the Injury risk in women (Legerlotz and Nobis). The authors discuss existing knowledge on the relationships between female hormones, musculoskeletal properties, neurophysiological changes and the risk to suffer from an injury. They also emphasize that a multitude of questions need to be answered, several contradictions to be solved, and several correlations to be explained (Legerlotz and Nobis).

Sichting and colleagues performed an explorative cross-sectional study in 40 identical twin pairs, investigating the effects of regular recreational exercise activities on Achilles tendon mechanical properties (Sichting et al.). The authors found a 28% greater Achilles tendon stiffness in physically active twins compared to their inactive twin. Furthermore, they found sport-specific adaptations, i.e., by demonstrating that the stiffness of tendons responded more to exercise activities with an “aerial phase” such as running and jumping (Sichting et al.).

Hernando et al. monitored renal function in 76 marathon finishers (14 females) starting from the day before the marathon until 192 h after completing the race. Recovery effects were investigated under 3 different conditions: total rest (REST), continuous running at their ventilatory threshold 1 (VT1) intensity (RUN), and elliptical workout at their VT1 intensity (ELLIPTICAL). Unexpectedly, the ELLIPTICAL group showed a significantly lower glomerular filtration rate compared to the RUN and the REST group.

## Air pollution and exercise

The known changes in climatic and environmental conditions, also related to the extreme diffusion of pollutants and increased average temperature, on the one hand, and the increasing outdoor sports practice on a large scale, on the other, require great attention to the physiological aspects of adaptations to exercise carried out in critical conditions, both in acute and chronic settings. The study of these interactions represents a major research challenge for the future, with fundamental predictable social implications.

In the present RT, You et al. explored potential effects on health when physical exercise (PE) is combined with exposure to air pollution (AP), by applying cluster, co-citation, and co-occurrence analysis using CiteSpace and VOSviewer software (You et al.). The research distribution, major topics, and relevant hotspots of the PE-AP field have been described by the knowledge-map strategy. At least with regard to the respiratory system, the first barrier of the body to defense against air pollutants, the benefits of exercise might be offset under AP conditions. Finally, the authors highlight a couple of research questions that should be considered by future studies (You et al.).

## Concluding remarks

Exercise physiology amazes for the enormous variety of research fields, each of which has a direct potential impact on the human’s quality of life.

Despite this heterogeneity a *trait d’union* is the intrinsic value of exercise in determining a plethora of physiological adaptations whose impact depend on the actual conditions and objectives.

From the role of polypill to fundamental determinant of the athletic performance, exercise represents a field of extreme interest, with transversal entanglements in the various scientific disciplines, from engineering to medical fields, and in its multidisciplinary nature is its beauty.

This will ensure that research in this area will not find moments of stalemate or reduction of interest on the part of those who dedicate their lives to research.
